# Critically appraised topic on adverse food reactions of companion animals (3): prevalence of cutaneous adverse food reactions in dogs and cats

**DOI:** 10.1186/s12917-017-0973-z

**Published:** 2017-02-15

**Authors:** Thierry Olivry, Ralf S. Mueller

**Affiliations:** 1 Department of Clinical Sciences, College of Veterinary Medicine, North Carolina State University, 1060 William Moore Drive, Raleigh, NC 27607 USA; 2 Medizinische Kleintierklinik, Centre for Clinical Veterinary Medicine, Ludwig Maximilian University, Veterinärstrasse 13, 80539 Munich, Germany

**Keywords:** Allergy, Atopic Dermatitis, Canine, Cat, Dog, Feline, Food Allergy, Itch, Pruritus

## Abstract

**Background:**

The prevalence of cutaneous adverse food reactions (CAFRs) in dogs and cats is not precisely known. This imprecision is likely due to the various populations that had been studied. Our objectives were to systematically review the literature to determine the prevalence of CAFRs among dogs and cats with pruritus and skin diseases.

**Results:**

We searched two databases for pertinent references on August 18, 2016. Among 490 and 220 articles respectively found in the Web of Science (Science Citation Index Expanded) and CAB Abstract databases, we selected 22 and nine articles that reported data usable for CAFR prevalence determination in dogs and cats, respectively. The prevalence of CAFR in dogs and cats was found to vary depending upon the type of diagnoses made. Among dogs presented to their veterinarian for any diagnosis, the prevalence was 1 to 2% and among those with skin diseases, it ranged between 0 and 24%. The range of CAFR prevalence was similar in dogs with pruritus (9 to 40%), those with any type of allergic skin disease (8 to 62%) and in dogs diagnosed with atopic dermatitis (9 to 50%). In cats presented to a university hospital, the prevalence of CAFR was less than 1% (0.2%), while it was fairly homogeneous in cats with skin diseases (range: 3 to 6%), but higher in cats with pruritus (12 to 21%) than in cats with allergic skin disease (5 to 13%).

**Conclusions:**

Among dogs and cats with pruritus and those suspected of allergic skin disease, the prevalence of CAFR is high enough to justify this syndrome to be ruled-out with a restriction (elimination)-provocation dietary trial. This must especially be considered in companion animals with nonseasonal pruritus or signs of allergic dermatitis.

**Electronic supplementary material:**

The online version of this article (doi:10.1186/s12917-017-0973-z) contains supplementary material, which is available to authorized users.

## Background

There is variability about the reported prevalence of cutaneous adverse food reactions (CAFRs) in dogs and cats. This heterogeneity of data might be caused by a combination of differences in the geographical populations studied, variability in animal groups in which the prevalence is reported and, perhaps, in the method of diagnosis of CAFR itself.

### Clinical scenario

You have two patients: a 1-year-old male intact West Highland white terrier and a 3-year-old female spayed Siamese cat. Both animals exhibit pruritus that manifests by year-round scratching. The dog also suffers from occasional episodes of urticaria, as well as bouts of soft mucus-containing stools. The cat has several patches of self-induced hair loss on the abdomen and medial thighs. You inform the owners of both patients that you suspect that all clinical signs might be caused by a reaction to their pet’s diet. The owners ask you how frequent this type of problem is.

### Structured question

What is the prevalence of CAFR among dogs and cats with pruritus or skin diseases?

### Search strategy

We searched the Web of Science (Science Citation Index Expanded) and CAB Abstract databases on August 18, 2016 using the following string: ((dog or dogs or canine) or (cat or cats or feline)) and (food or diet*) and (atop* or allerg* or reaction*) and (prurit* or cutan* or skin) not (human* or adult* or child*). We limited the search to journal articles published from 1980 to present; there were no language restrictions.

### Identified evidence

Our literature search identified 490 and 220 articles in the CAB Abstract and Web of Science databases, respectively. Citations were initially assessed for the identification of articles reporting original information; review papers were not considered further. Abstracts were then screened and potentially relevant papers were read in full. The bibliography of these articles was examined further for additional pertinent citations.

Altogether, we selected 28 papers that provided usable information [1–28]. Twenty-seven articles were identified from the search of the CAB abstract database, while 18 of these 27 papers (67%) were also found in the Web of Science archives; none was uniquely detected in the Web of Science query, while one additional publication was identified from scanning the references of selected articles [14]. There were nine studies reporting information on the prevalence of CAFR in cats [1, 3, 5, 10, 22, 24–27] and 22 on that in dogs [1–4, 6–21, 23, 28]; three reported data usable for both dogs and cats [1, 3, 10]. Studies were reported from 1990 [1] to 2015 [28]. All papers were in English except for one each in French [3], Dutch [4], German [9], Italian [13] and Portuguese [18].

### Evaluation of evidence

The selected articles reported information from small animal patients from all over the world: cats came from Australia [26, 27], Canada [1, 3], New Zealand [5], the UK [10], the USA [24, 25] or from a worldwide survey [22]. Dogs with CAFR had been diagnosed in Brazil [18, 19, 28], Canada [1, 3], the Czech Republic [16], Hungary [14], Iran [23], Italy [13, 20], the Netherlands and Belgium [4, 7], Slovenia [15], Switzerland [9, 17], Sweden [12], the UK [6, 8, 10, 11] and the USA [2]; there was also a large worldwide survey [21]. Only two articles contained reviews of diagnoses made in general veterinary practices [10, 12], while all other reports were from patients seen at university or private specialty clinics.

The method of diagnosis of CAFR was not specified in three surveys [1, 10, 18], while, in all other reports, the diagnosis was made after observing a reduction of pruritus manifestations after feeding an elimination diet lasting most often between 6 and 8 weeks. In all but four studies [3, 12, 14, 28], this elimination diet was followed by a challenge with offending allergens. Importantly, in only four articles was an elimination diet performed in the entire population of study patients.

**Fig. 1:**
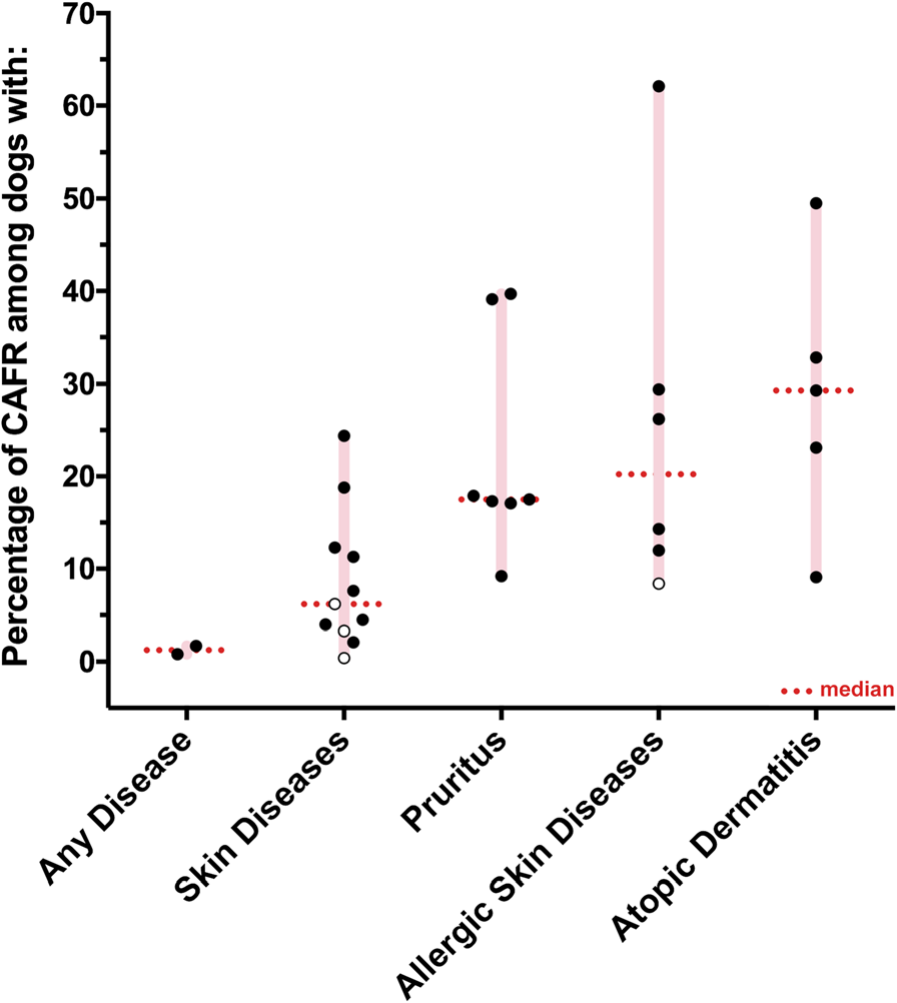
Prevalence of CAFRs among dogs with various conditions. Open circles correspond to the three studies in which the method of diagnosis of CAFR was not specified [1, 10, 18]

**Fig. 2:**
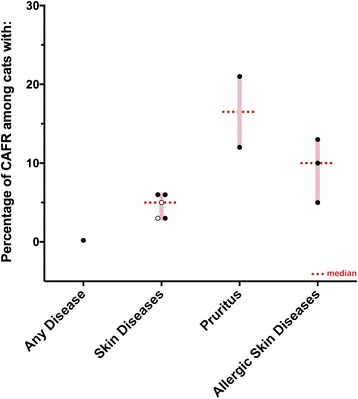
Prevalence of CAFRs among cats with various conditions. Open circles correspond to the two studies in which the method of diagnosis of CAFR was not specified [1, 10, 18]

The prevalence of CAFRs in dogs and cats was found to vary depending upon the type of diagnosis made. In dogs (Fig. 1), the prevalence of CAFRs was low among dogs presented to their veterinarian for any diagnosis (1 to 2%) or among those with skin diseases (median: 6%; range: 0 to 24%). Furthermore, ranges of reported prevalence of CAFR overlapped between dogs with pruritus (median: 18%; range: 9 to 40%), those with any type of allergic skin disease (median: 20%; range: 8 to 62%) and dogs with skin lesions suggestive of atopic dermatitis (median: 29%; range: 9 to 50%) (Fig. 1; Additional file 1). A similar pattern was found in feline patients (Fig. 2). In cats presented to a university hospital [24], the prevalence of CAFR was reported to be very low (0.2%), while it was fairly homogeneous in cats with skin diseases (median: 5%; range: 3 to 6%); it was higher in cats with pruritus (12 and 21%) than in cats with allergic skin disease (median: 10%; range: 5 to 13%) (Fig. 2; Additional file 2). We attribute the latter observation to cats occasionally manifesting a CAFR as pruritus without visible dermatitis. Altogether, there were not enough data to compare the prevalence of CAFR in dogs and cats from different geographical locations.

As in most summaries incorporating results from studies performed at different times and institutions, the main limitation of this review is the likely variability of methods or criteria used to make the diagnosis of CAFR. A similar inconsistency probably also existed in the way atopic dermatitis was diagnosed between studies. Whenever details were provided, however, CAFRs and AD were diagnosed according to accepted standards at the time of publication. Importantly, in all but four studies [7, 8, 11, 17], not all animals from the reported population (e.g. dogs with any or skin diseases) had been subjected to an elimination diet. This lack of systematic dietary testing likely led to a lower prevalence of CAFR reported in articles where the diet change was not made in all pets.

## Conclusion and implication for practitioners

Our review of the existing evidence suggests that the prevalence of CAFRs in dogs and cats varies depending upon the population in which it is calculated. Despite the likely heterogeneity existing between methods of diagnosis, the prevalence of CAFRs in companion animals appears somewhat similar. Among dogs and cats with any disease, skin disease, pruritus or allergic skin disease, the median prevalence of CAFR is less than 1%, about 5%, between 15 to 20% and 10 to 25%, respectively; it is also estimated to be around one third of dogs with atopic dermatitis.

## Additional files

Additional file 1: Specific data on the prevalence of CAFRs among dogs with various conditions. (XLSX 45 kb)
Additional file 2: Specific data on the prevalence of CAFRs among cats with various conditions. (XLSX 40 kb)

## Abbreviations

CAFR: Cutaneous adverse food reaction
CAT: Critically appraised topic
